# New and interesting findings of scarab beetles (Coleoptera, Scarabaeoidea) from Tajikistan

**DOI:** 10.3897/zookeys.1003.55457

**Published:** 2020-12-14

**Authors:** Adam Byk, Andrzej Matusiak, Artur Taszakowski, Wojciech T. Szczepański, Marcin Walczak, Marek Bunalski, Lech Karpiński

**Affiliations:** 1 Department of Forest Protection, Institute of Forest Sciences, Warsaw University of Life Sciences – SGGW, Nowoursynowska 159/34, PL-02-776, Warsaw, Poland Warsaw University of Life Sciences – SGGW Warsaw Poland; 2 Odyńca 69/31, PL-02-664, Warsaw, Poland Unaffiliated Warsaw Poland; 3 Institute of Biology, Biotechnology and Environmental Protection, Faculty of Natural Sciences, University of Silesia in Katowice, Bankowa 9, PL-40-007, Katowice, Poland University of Silesia in Katowice Katowice Poland; 4 Silesian Entomological Society, Jana III Sobieskiego 2, PL-41-902, Bytom, Poland Silesian Entomological Society Bytom Poland; 5 Department of Entomology and Environmental Protection, University of Life Sciences, Dąbrowskiego 159, PL-60-594, Poznań, Poland University of Life Sciences Poznań Poland; 6 Museum and Institute of Zoology, Polish Academy of Sciences, Wilcza 64, PL-00-679, Warsaw, Poland Museum and Institute of Zoology, Polish Academy of Sciences Warsaw Poland

**Keywords:** *Euonthophagus koshantschikoffi*, Geotrupidae, Glaphyridae, Hybosoridae, new records, *Rhyssemodes transcaspicus*, Scarabaeidae

## Abstract

We report on new findings of nearly 50 species that represent four families of the superfamily Scarabaeoidea, which were collected during an expedition to western Tajikistan that was carried out in June and July 2014. *Rhyssemodes transcaspicus* Rakovič, 1982 is recorded from the country for the first time. Moreover, we describe and illustrate the differences in the external morphology between *Euonthophagus gibbosus* (Scriba, 1790) and *E. koshantschikoffi* (Reitter, 1891), the latter of which has a doubtful systematic position. In the collected material of approximately 1,000 specimens, more than 90% of the species and 95% of the individuals belong to the family Scarabaeidae. The other species represent the families Geotrupidae, Glaphyridae, and Hybosoridae.

## Introduction

The first comprehensive study that contained information on the scarabaeoid beetle fauna of Tajikistan was presented by Medvedev and Lopatin (1961), who reported on 183 species in the country but provided scant data on habitats, biology, and distribution. More information on the local Scarabaeoidea of the subfamilies Melolonthinae, Rutelinae, and Dynastinae, as well as on the family Glaphyridae, can be found in the earlier monographs of the notable “Fauna SSSR” series (Medvedev 1949, 1951, 1952, 1960) and on the subfamilies Cetoninae and Valginae in the later continuation of this series (Medvedev 1964). These works also contain identification keys to species. Additionally, some information on the scarabaeoid beetles of the subfamily Aphodiinae that occur in Tajikistan was included in the studies of Protzenko (1968), Nikritin (1973), and Nikolajev (1987). Nikolajev (2003) published another work of a great importance for recognising the fauna of the family Geotrupidae; the paper included species of the genus *Lethrus* Scopoli, 1777 (Lethrinae), which constitutes a significant share in the local beetle fauna. Three years later, an extensive monograph containing comprehensive information on the subfamily Scarabaeinae (Scarabaeini, Gymnopleurini, Onthophagini, Onitini) of Russia and adjacent countries, including Tajikistan, was released (Kabakov 2006). According to the latest edition of the Catalogue of Palaearctic Coleoptera, 274 species of scarabaeoid beetles are currently known from Tajikistan (Löbl and Löbl 2016).

The majority of the works on the Scarabaeoidea of Tajikistan were published in the last century. They treat the scarabaeoid Tajik fauna in the context of areas that often reach far beyond the current boundaries of the country, and sometimes even beyond the commonly accepted borders of Central Asia. Consequently, many records have a rather general character without any precise locality data. Therefore, we aim to supplement the current information on the distribution of the taxa of the superfamily Scarabaeoidea in the country. An additional goal of our study is to gather and disseminate information contained in valuable publications that are, however, difficult to access and were usually published exclusively in Russian.

## Material and methods

Tajikistan is a relatively small intra-continental country that is situated at the boundary of the subtropical and temperate climatic zones. It is located in the mountain desert zone of the Eurasian continent, in the southern part of Central Asia. In this region, diverse ecosystems such as deserts, steppes, conifer forests, mixed mountain forests, and high-mountain deserts are widely represented. The formation of this unique biological diversity in the country, which counts numerous endemic and relict species, is due to the varied mountain climatic conditions and historical natural processes (Safarov 2003).

**Figure 1.**
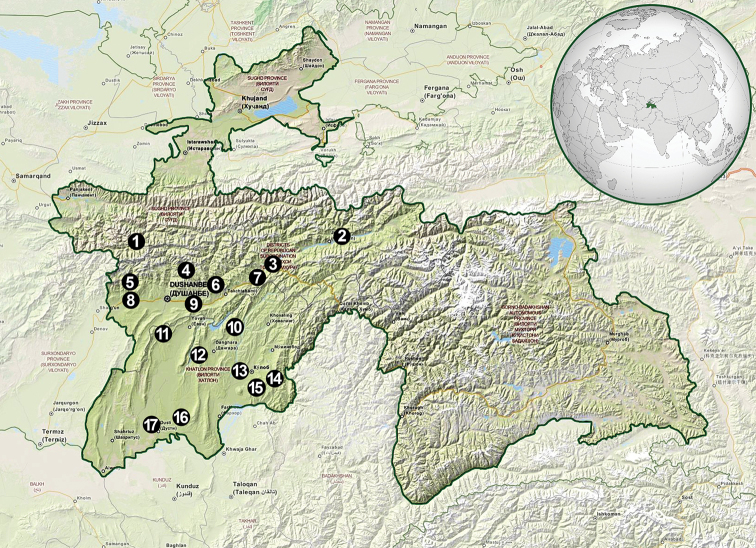
Research plots in the western part of Tajikistan **1** Iskanderkul **2** Tojikobod **3** Gharm **4** Safed Dara (Takob) **5** Kuran, Karatag river valley **6** Romit **7** Komsomolabad and Nurobod **8** Shahrinav **9** Arykboshi **10** Chavrok and Kangurt **11** Dohanaklik **12** Ganchi **13** W of Kulob, Vose–Kulob road **14** Shurroabad **15** Sarichashma **16** Novabad **17** Jilikul (OpenStreetMap contributors).

An entomological expedition, which consisted of four scientists from the former Department of Zoology, University of Silesia in Katowice (Poland), was undertaken over 25 days from 24 June to 18 July 2014. During the research, several field surveys in various locations in the western part of Tajikistan were conducted (Fig. 1). The most extensive studies were carried out in the central part of the region and in the south-western part of the country along the Afghanistan border. Our investigations were conducted in several research plots including the villages or environs of Arykboshi, Dushanbe, Dohanaklik, Ganchi, Gharm, Iskanderkul, Jilikul, Kangurt, Karatag, Komsomolabad, Kulob, Nurobod, Romit, Sarichashma, Shahrinav, Shurroabad, Takob, Tojikobod, and Vose (Table 1).

**Table 1.** Collection sites of the scarabaeoid beetles in Tajikistan (2014).

No.	Locality	Geographical coordinates	Altitude [m a.s.l.]	Date of collection
1.	Iskanderkul [Искандарkӯл] (Fig. 2A)	39°05'04.1"N, 68°22'03.2"E	2300	18 Jul. 2014
2.	Tojikobod [Тоҷикобод] (Fig. 2B)	39°05'34.1"N, 70°51'45.2"E	2225	13 Jul. 2014
3.	Gharm [Ғарм] (Fig. 2C)	39°01'11.1"N, 70°22'07.6"E	1330	14 Jul. 2014
4.	Safed Dara [Caфeд Дapa] = Takob (Taкoб) (Fig. 2D)	38°51'30.7"N, 68°59'58.7"E	2300	27–28 Jun. 2014
		38°49'27.9"N, 68°56'10.4"E	1850	8–10 Jul. 2014
5.	Karatag river valley, near Kuran [Куран] (Fig. 2F)	38°41'44.8"N, 68°22'05.1"E	1060	30 Jun.–1 Jul. 2014
				17 Jul. 2014
6.	Romit [Ромит] (Fig. 2G)	38°46'19.3"N, 69°16'58.5"E	1285	26–27 Jun. 2014
7.	Komsomolabad [Дарбанд] (Fig. 2H)	38°51'50.2"N, 69°56'32.0"E	1160	11–12 Jul. 2014
	Nurobod [Нуробод] (Fig. 3A)	38°47'45.2"N, 69°51'32.6"E	1215	11 Jul. 2014
8.	Shahrinav [Шаҳринав]	38°36'04.1"N, 68°19'36.0"E	870	1 Jul. 2014
9.	Arykboshi [Арыкбошӣ]	38°34'39.3"N, 69°04'03.7"E	905	28 Jun., 2 Jul. 2014
10.	Kangurt [Kангурт] env.	38°11'57.7"N, 69°33'18.7"E	1065	5 Jul. 2014
	Chavrok [Чаврок], N of Kangurt (Fig. 3B)	38°18'07.9"N, 69°32'51.5"E	1215	5 Jul. 2014
11.	Dohanaklik [Даханакиик] (Fig. 3C)	38°13'50.4"N, 68°40'23.0"E	860	16 Jul. 2014
12.	Ganchi [Сафедсанг]	38°00'08.4"N, 69°09'39.4"E	765	25 Jun. 2014
13.	W of Kulob [Куляб], Vose–Kulob road (Fig. 3D)	37°54'37.0"N, 69°42'41.1"E	525	3 Jul. 2014
		37°54'56.3"N, 69°42'53.6"E	535	3 Jul. 2014
14.	Shurroabad [Ноҳияи Шӯрообод] (Fig. 3E)	37°49'16.1"N, 70°03'20.4"E	2150	4 Jul. 2014
15.	Sarichashma [Сары-Чашма]	37°44'47.6"N, 69°46'19.0"E	920	25 Jun. 2014
15.	Novabad [Новабад] (Fig. 3G)	37°30'14.3"N, 68°53'20.3"E	580	16–17 Jul. 2014
17.	Jilikul [Ҷиликӯл] (Fig. 3H)	37°27'05.5"N, 68°31'16.7"E	330	24 Jun. 2014

**Figure 2.**
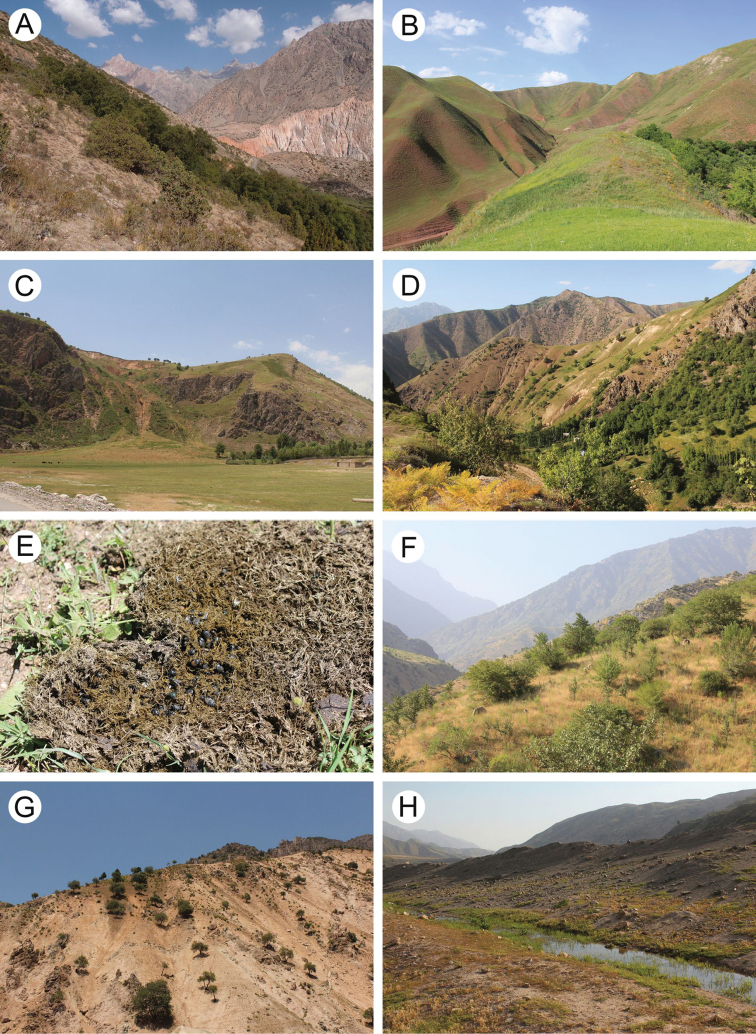
Typical landscapes in Tajikistan, habitats of scarabeoid beetles **A** mountain slope covered with shrubs including *Juniperus* in Iskanderkul environs **B** mountain meadow in Tojikobod environs **C** mountain slopes in Gharm environs **D** mountain meadow overgrown by *Prangos* and *Ferula* in Safed Dara environs **E** swarm of *Onthophagus* beetles in cow dung, Safed Dara environs **F** mountain slope covered with shrubs in Karatag environs **G** steep slope covered with single trees in Romit environs **H** watercourse near a pasture in Komsomolabad environs.

**Figure 3.**
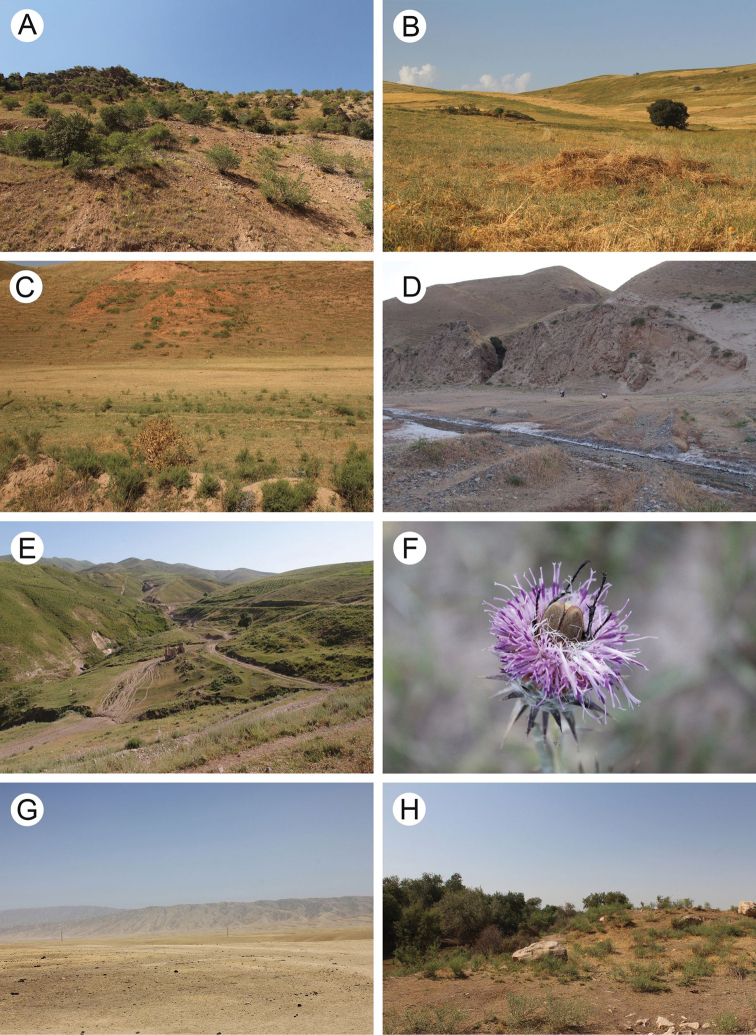
Typical landscapes in Tajikistan, habitats of scarabeoid beetles **A** lush shrub vegetation in Nurobod environs **B** grasslands in Chavrok environs **C** grasslands in Dohanaklik environs **D** grazed slopes in Kulob environs **E** grassy hills in Shurroabad environs **F***Glaphyrus turkestanicus* feeding in flower cup, Shurroabad environs **G** semi-desert with lots of dung in Novabad environs **H** bank of Vakhsh River overgrown with tamarisk shrubs in Jilikul environs.

The area covered by our study includes several different nature ecosystems such as alpine meadows, mesophilic shrubs, shrub steppes, broad-leaf forests, and tugay, as well as agroecosystems such as gardens, orchards, fields, and pastures (Figs 2, 3). Various field methods were used because scarabaeoid beetles comprise species with a very diverse biology. However, because coprophagy is the main type of feeding behaviour in this group, most of the sampling took place in pastures and semi-desert areas where dung of domestic and wild animals was examined (Fig. 2E). Additionally, beetles were attracted to the artificial light sources during the night.

Beetles were photographed in their habitats with Canon EOS 550D, Canon EOS 600D, and Olympus XZ-1 cameras. Mounted specimens were imaged using a Leica M205C stereomicroscope with Leica LED5000 HDI high-diffuse dome illumination and equipped with a Leica DFC495 digital camera. The images that were produced were stacked, aligned, and combined using Leica application suite v. 4.9.0 software. The images selected were cleaned, retouched, and arranged into figures using Adobe Photoshop CS6 software. The geographical coordinates were recorded using a Garmin Oregon 550T 3-Inch Handheld GPS Navigator. For each specimen that was collected, the exact location, altitude, date, and the names of the collectors are given.

All the specimens listed below were collected by Artur Taszakowski (AT), Lech Karpiński (LK), Marcin Walczak (MW), and Wojciech T. Szczepański (WTS). Taxa were identified by Adam Byk (AB), Andrzej Matusiak (AM), and Marek Bunalski (MB). The specimens are preserved in the entomological collection of the Department of Natural History of the Upper Silesian Museum, Bytom, Poland (USMB) and in the collections of the authors. The systematic arrangement and nomenclature were adopted from the Catalogue of Palaearctic Coleoptera (Löbl and Löbl 2016).

## Results

As a result of this study, about 950 beetles belonging to 48 species in four families of the superfamily Scarabaeoidea were collected: Geotrupidae (1 sp.), Glaphyridae (2 spp.), Hybosoridae (1 sp.), and Scarabaeidae (44 spp.), including Scarabaeinae (17 spp.), Aphodiinae (12 spp.), Melolonthinae (5 spp.), Cetoniinae (5 spp.), Rutelinae (3 spp.), and Dynastinae (2 spp.). The list of the recorded taxa along with their new localities is presented below.

### Family Geotrupidae Latreille, 1802

#### Subfamily Lethrinae Oken, 1843

**Lethrus (Mesolethrus) sp.**

Karatag, 17 Jul. 2014, 1058 m a.s.l., [MW] – 1 ex. (♀);

Shurroabad, 4 Jul. 2014, 2152 m a.s.l., in dung, [WS] – 1 ex. (♀).

**Remarks.** Three species from the subgenus Mesolethrus Nikolajev, 2003 were recorded from Tajikistan, and another two from the neighboring Uzbekistan (Bagaturov and Nikolajev 2015). Females of these species are extremely similar and thus almost unidentifiable. The determination was additionally hindered by significant degree of body damage of our specimens.

### Family Hybosoridae Erichson, 1847

#### Subfamily Hybosorinae Erichson, 1847

***Hybosorus illigeri* Reiche, 1853**

Chavrok, N of Kangurt, 5 Jul. 2014, 1217 m a.s.l., at light, [WS] – 1 ex., [MW] – 2 exx.

### Family Glaphyridae Macleay, 1819

#### Subfamily Amphicominae Blanchard, 1845

**Eulasia (Solskiola) analis (Solsky, 1876)**

Romit, 27 Jun. 2014, 1283 m a.s.l., [WS] – 1 ex.;

Takob, 27 Jun. 2014, 2298 m a.s.l., [LK] – 1 ex.

**Remarks.** This species occurs in Uzbekistan, southern Turkmenistan, northeastern Iran, and northern Afghanistan. In Tajikistan, it was reported from a number of localities: Mumilabad, Kulab, Langar, Darvaz (Medvedev 1960). This is an early spring species, which is usually found from March to May (Medvedev 1960; Medvedev and Lopatin 1961).

We collected it at the end of June in high mountain meadows near Romit and Takob. Two individuals were found on flowers of herbaceous plants.

#### Subfamily Glaphyrinae W.S. Macleay, 1819

**Glaphyrus (Eoglaphyrus) turkestanicus Semenov, 1889**

Shurroabad, 4 Jul. 2014, 2152 m a.s.l., mountain meadows, pastures, [AT] – 1 ex.

**Remarks.***Glaphyrus turkestanicus* is distributed in Uzbekistan, Afghanistan (Medvedev 1960), and Tajikistan, where it was recorded mainly from the northern part of the country (Oburdon, Artuch) (Medvedev and Lopatin 1961).

We collected a single male of this species in a mountain meadow near Shurroabad. The beetle was found inside a calyx of *Carduus* sp. (Fig. 3F).

### Family Scarabaeidae Latreille, 1802

#### Subfamily Aphodiinae Leach, 1815

***Acanthobodilus immundus* (Creutzer, 1799)**

W of Kulob, 3 Jul. 2014, 526 m a.s.l., at light, [AT] – 5 exx.;

W of Kulob, 3 Jul. 2014, 537 m a.s.l., at light, [WS] – 1 ex.;

Kangurt, 5 Jul. 2014, 1066 m a.s.l., in dung, [WS] – 1 ex., at light, [AT] – 2 exx., in dung, [MW] – 3 exx.;

Chavrok, N of Kangurt, 5 Jul. 2014, 1217 m a.s.l., at light, [WS] – 4 exx.;

Komsomolabad, 11 Jul. 2014, 1160 m a.s.l., in dung, [WS] – 1 ex.;

Dohanaklik, 16 Jul. 2014, 862 m a.s.l., in dung, [WS] – 3 exx.;

Novabad, 17 Jul. 2014, 580 m a.s.l., desert, semi-desert, in cow dung, [AT] – 1 ex., [MW] – 4 exx.

***Acrossus luridus* (Fabricius, 1775)**

Takob, 28 Jun. 2014, 2300 m a.s.l., [WS] – 2 exx.

***Aphodius pedellus* De Geer, 1774**

Romit, 26 Jun. 2014, 1250 m a.s.l., [WS] – 8 exx.;

Karatag, 17 Jul. 2014, 1058 m a.s.l., in cow dung, [AT] – 1 ex., [MW] – 1 ex.

***Bodilus lugens* (Creutzer, 1799)**

Karatag, 1058 m a.s.l., 30 Jun. 2014, in dung, [MW] – 5 exx., 17 Jul. 2014, in cow dung, [AT] – 3 exx., [MW] – 2 exx., shrubs, [AT] – 1 exx.;

Kangurt, 5 Jul. 2014, 1066 m a.s.l., agrocenoses, at light, [AT] – 2 exx., [MW] – 3 exx.;

Chavrok, N of Kangurt, 5 Jul. 2014, 1217 m a.s.l., at light, [WS] – 2 exx.;

Novabad, 17 Jul. 2014, 580 m a.s.l., desert, semi-desert, in cow dung, [AT] – 1 ex.

***Colobopterus erraticus* (Linnaeus, 1758)**

Romit, 27 Jun. 2014, 1250 m a.s.l., [WS] – 2 exx.;

Karatag, 30 Jun. 2014, 1058 m a.s.l., in dung, [WS] – 1 ex., [MW] – 6 exx.;

Komsomolabad, 11 Jul. 2014, 1160 m a.s.l., in dung, [WS] – 1 ex., [MW] – 1 ex.

***Esymus pusillus pusillus* (Herbst, 1789)**

Takob, 28 Jun. 2014, 2300 m a.s.l., [WS] – 2 exx., [MW] – 3 exx.;

Arykboshi, 2 Jul. 2014, 906 m a.s.l., [WS] – 1 ex., [MW] – 3 exx.

***Eudolus quadriguttatus* (Herbst, 1783)**

Kangurt, 5 Jul. 2014, 1066 m a.s.l., agrocenoses, at light, [AT] – 1 ex.;

Chavrok, N of Kangurt, 5 Jul. 2014, 1217 m a.s.l., at light, [WS] – 3 exx.

***Labarrus lividus* (Olivier, 1789)**

Karatag, 17 Jul. 2014, 1058 m a.s.l., at light, [AT] – 1 ex.;

Kangurt, 5 Jul. 2014, 1066 m a.s.l., agrocenoses, at light, [AT] – 1 ex.;

Chavrok, N of Kangurt, 5 Jul. 2014, 1217 m a.s.l., at light, [WS] – 4 exx.

***Neocalaphodius moestus* (Fabricius, 1801)**

Fig. 4A

Jilikul, 24 Jun. 2014, 332 m a.s.l., at light, [WS] – 5 exx.;

Arykboshi, 2 Jul. 2014, 906 m a.s.l., at light, [WS] – 1 ex., [MW] – 2 exx.;

Karatag, 30 Jun. 2014, 1058 m a.s.l., at light, [AT] – 1 ex., [MW] – 2 exx.;

W of Kulob, 3 Jul. 2014, 526 m a.s.l., at light, [AT] – 5 exx.;

W of Kulob, 3 Jul. 2014, 537 m a.s.l., at light, [WS] – 1 ex.;

Chavrok, N of Kangurt, 5 Jul. 2014, 1217 m a.s.l., at light, [WS] – 1 ex.;

Dohanaklik, 16 Jul. 2014, 862 m a.s.l., in dung, [WS] – 3 exx.;

Novabad, 17 Jul. 2014, 580 m a.s.l., desert, semi-desert, in cow dung, [AT] – 1 ex., [MW] – 1 ex.

**Figure 4.**
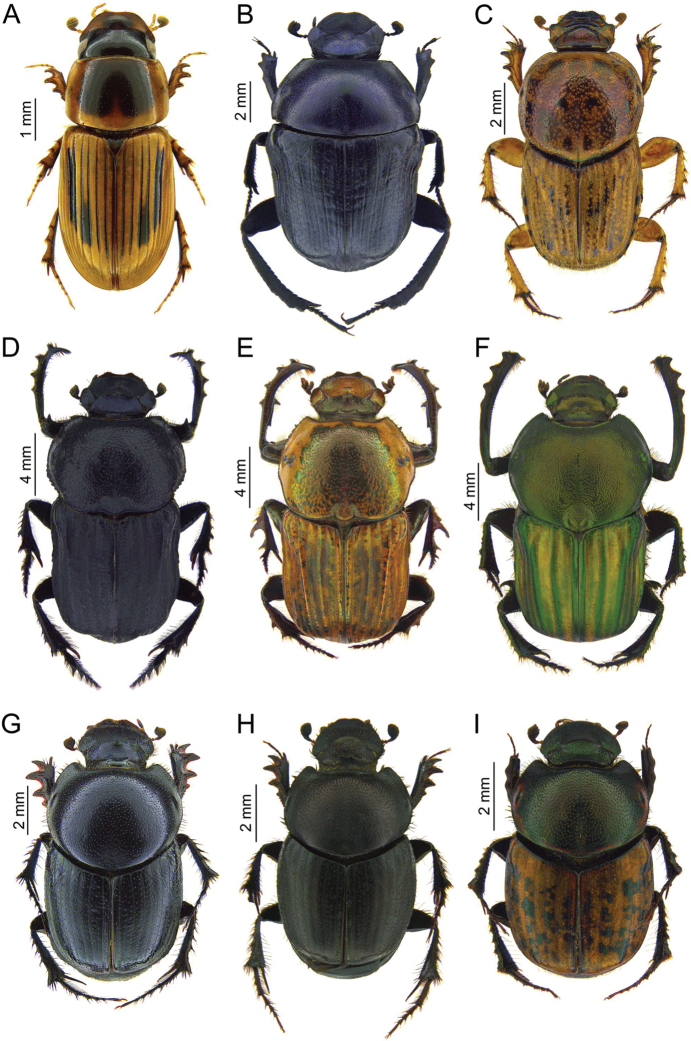
Photos of Scarabaeidae specimens collected during the expedition to Tajikistan in 2014 **A***Neocalaphodius moestus***B***Gymnopleurus aciculatus***C***Euoniticellus pallipes***D***Cheironitis haroldi*, male **E***Cheironitis pamphilus*, male **F***Onitis humerosus*, male **G***Euonthophagus sulcicollis*, female **H***Onthophagus sibiricus*, female **I***Onthophagus haroldi*.

***Planolinellus vittatus* (Say, 1825)**

Kangurt, 5 Jul. 2014, 1066 m a.s.l., in dung, [MW] – 2 exx.

***Rhyssemodes transcaspicus* Rakovič, 1982**

Fig. 5

Jilikul, 24 Jun. 2014, 332 m a.s.l., at light, [WS] – 6 exx., [MW] – 1 ex.;

W of Kulob, 3 Jul. 2014, 537 m a.s.l., at light, [WS] – 7 exx., [MW] – 1 ex.;

Chavrok, N of Kangurt, 5 Jul. 2014, 1217 m a.s.l., at light, [WS] – 1 ex.

**Remarks.** This species has been reported from Uzbekistan and the southern territory of European Russia. According to Rakovič and Král (2015), its presence in Uzbekistan is confirmed by the holotype’s label: “Golodnaya Step” (about 120 km southeast of Tashkent). The presence of *Rh. transcaspicus* in Astrakhan Province and Kalmykia, Russia, was documented by Shokhin (2007) and Shokhin et al. (2014). Although *Rh. transcaspicus* had been synonymised with *Rhyssemodes tenuisculptus* Reitter, 1892 by Nikolajev (1987), it was restored 15 years later by Shokhin (2002).

We collected 16 individuals of *Rh. transcaspicus* at three different sites in the western part of the country, about 300 km south of its type locality. All individuals were found near human settlements with semi-arid (environs of Jilikul) and farmland (environs of Kulob and Chavrok) habitats in the immediate vicinity. In all cases, imagines were attracted to UV light.

**Figure 5.**
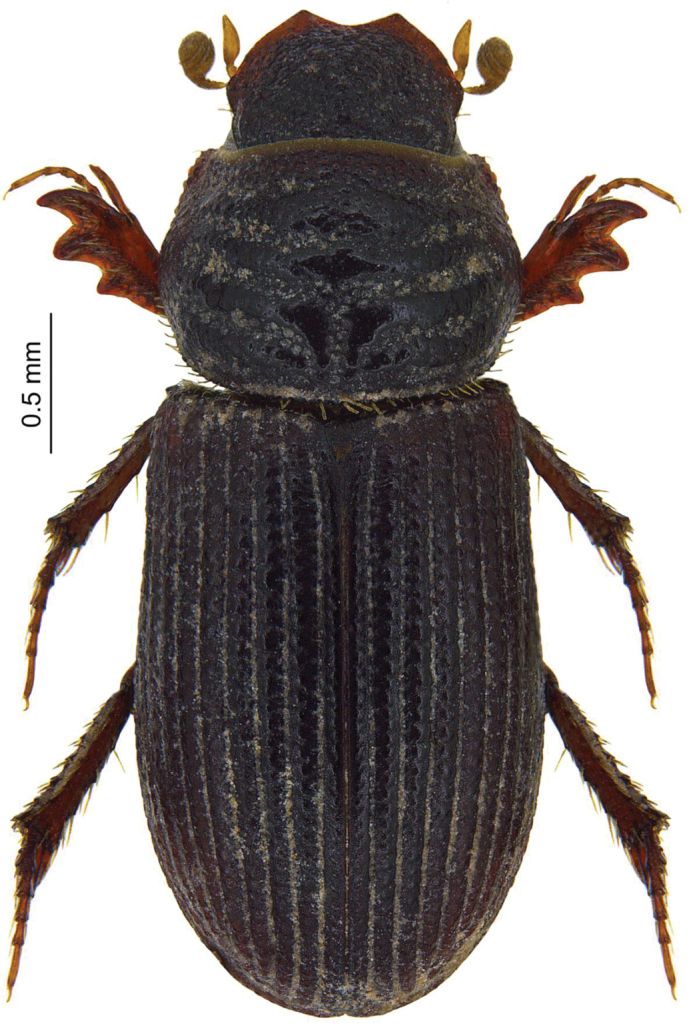
*Rhyssemodes transcaspicus* Rakovič, 1982, species new for the Tajik fauna.

This is the first record for Tajikistan. The material was additionally verified by Łukasz Minkina (Poland).

***Rhyssemus germanus* (Linnaeus, 1767)**

W of Kulob, 3 Jul. 2014, 526 m a.s.l., at light, [AT] – 1 ex.;

W of Kulob, 3 Jul. 2014, 537 m a.s.l., at light, [WS] – 1 ex.

#### Subfamily Scarabaeinae Latreille, 1802

***Gymnopleurus aciculatus* Gebler, 1841**

Fig. 4B

Karatag, 17 Jul. 2014, 1058 m a.s.l., in cow dung, [MW] – 5 exx.;

W of Kulob, 3 Jul. 2014, 526 m a.s.l., in dung, [WS] – 1 ex., [MW] – 5 exx.;

Shurroabad, 4 Jul. 2014, 2152 m a.s.l., in dung, [WS] – 5 exx., [AT] – 10 exx.;

Kangurt, 5 Jul. 2014, 1066 m a.s.l., in dung, [WS] – 3 exx., [MW] – 5 exx.;

Novabad, 17 Jul. 2014, 580 m a.s.l., desert, semi-desert, in cow dung, [MW] – 2 exx.

***Euoniticellus fulvus* (Goeze, 1777)**

Sarichashma, 25 Jun. 2014, 921 m a.s.l., in dung, [WS] – 5 exx.;

Romit, 26 Jun. 2014, 1250 m a.s.l., [WS] – 2 exx.;

Karatag, 30 Jun. 2014, 1058 m a.s.l., in cow dung, [MW] – 6 exx., 17 Jul. 2014, [AT] – 18 exx., [MW] – 14 exx.;

W of Kulob, 3 Jul. 2014, 526 m a.s.l., in dung, [WS] – 2 exx., [MW] – 13 exx., [AT] – 1 ex.;

Shurroabad, 4 Jul. 2014, 2152 m a.s.l., in dung, [WS] – 1 ex., [AT] – 1 ex.;

Komsomolabad, 11 Jul. 2014, 1160 m a.s.l., in dung [WS] – 8 exx., [MW] – 21 exx.;

Gharm, 14 Jul. 2014, in dung, [WS] – 6 exx.;

Dohanaklik, 16 Jul. 2014, 862 m a.s.l., in dung, [WS] – 6 exx., [AT] – 1 ex.;

Novabad, 17 Jul. 2014, 580 m a.s.l., desert, semi-desert, in cow dung, [AT] – 8 exx., [MW] – 3 exx.

***Euoniticellus pallipes* (Fabricius, 1781)**

Fig. 4C

Karatag, 30 Jun. 2014, 1058 m a.s.l., in cow dung, [MW] – 1 ex.;

W of Kulob, 3 Jul. 2014, 526 m a.s.l., in dung, [WS] – 14 exx., [MW] – 2 exx., [AT] – 1 ex.;

Kangurt, 5 Jul. 2014, 1066 m a.s.l., in dung, [WS] – 1 ex.;

Dohanaklik, 16 Jul. 2014, 862 m a.s.l., in dung, [WS] – 3 exx.;

Novabad, 17 Jul. 2014, 580 m a.s.l., [MW] – 1 ex.

***Cheironitis haroldi* (Ballion, 1871)**

Fig. 4D

Karatag, 30 Jun. 2014, 1058 m a.s.l., [MW] – 3 exx., 17 Jul. 2014, [MW] – 4 exx., [AT] – 2 exx.;

W of Kulob, 3 Jul. 2014, 526 m a.s.l., in dung, [MW] – 5 exx.;

Shurroabad, 4 Jul. 2014, 2152 m a.s.l., in dung, [WS] – 16 exx., [AT] – 2 exx.;

Novabad, 16 Jul. 2014, 580 m a.s.l., [MW] – 6 exx.

***Cheironitis pamphilus* (Menetries, 1849)**

Fig. 4E

Karatag, 30 Jun. 2014, 1058 m a.s.l., [AT] – 2 exx.;

W of Kulob, 3 Jul. 2014, 526 m a.s.l., in cow dung, [WS] – 1 ex., [MW] – 1 ex.;

Dohanaklik, 16 Jul. 2014, 862 m a.s.l., [WS] – 6 exx., [AT] – 2 exx.;

Novabad, 16 Jul. 2014, 580 m a.s.l., in horse dung, [WS] – 1 ex., [MW] – 2 exx., [AT] – 2 exx.

**Remarks.** This is a widely distributed species known from the southern part of the European territory of Russia, Georgia, Armenia, Azerbaijan, Iran, and Afghanistan; it is also widespread in Central Asia: Turkmenistan, Uzbekistan, and Tajikistan. Moreover, it was reported from Syria, Turkey, and Greece (Kabakov 2006), as well as from Cyprus, Lebanon, and Israel (Bezděk 2016c). In Tajikistan, it has been recorded from numerous localities, such as Dushanbe, Gissarskiy Khrebet, Vakhshkiy Khrebet, Khoviling, and Darvaz (Medvedev and Lopatin 1961).

We found most individuals in cow and horse dung in warm and dry meadows at altitudes from 500 to 1050 m a.s.l.

***Onitis humerosus* (Pallas, 1771)**

Fig. 4F

Shurroabad, 4 Jul. 2014, 2152 m a.s.l., in dung, [WS] – 2 exx.

***Euonthophagus amyntas subviolaceus* Ménétriés, 1832**

Takob, 9 Jul. 2014, 1850 m a.s.l., in dung, [WS] – 22 exx., [MW] – 12 exx.;

Arykboshi, 2 Jul. 2014, [MW] – 2 exx.;

Karatag, 30 Jun. 2014, 1058 m a.s.l., [MW] – 3 exx., 17 Jul. 2014, [AT] – 2 exx.;

W of Kulob, 3 Jul. 2014, 526 m a.s.l., in dung, [MW] – 3 exx.;

Shurroabad, 4 Jul. 2014, 2152 m a.s.l., in dung, [WS] – 6 exx., [AT] – 1 ex.

***Euonthophagus koshantschikoffi* (Reitter, 1891)**

Takob, 9 Jul. 2014, 1850 m a.s.l., in dung, [WS] – 21 exx., [MW] – 7 exx.;

Arykboshi, 2 Jul. 2014, [MW] – 2 exx.;

Karatag, 17 Jul. 2014, 1058 m a.s.l., [AT] – 4 exx., [MW] – 8 exx.;

W of Kulob, 3 Jul. 2014, 526 m a.s.l., in dung, [WS] – 3 exx., [MW] – 4 exx.;

Shurroabad, 4 Jul. 2014, 2152 m a.s.l., in dung, [WS] – 3 exx., [AT] – 2 exx.;

Novabad, 16 Jul. 2014, 580 m a.s.l., [MW] – 1 ex.

**Remarks.** We collected a series of individuals belonging to *E. koshantschikoffi*, a taxon with a uncertain systematic position. This species was described from the environs of Tashkent, Uzbekistan, under the name *Onthophagus koshantschikoffi* Reitter, 1891, and its range is limited to Central Asia. In 1972, this species was recognised as a junior synonym of *Onthophagus gibbosus* (Scriba, 1790), a much more widely distributed species which was described from southern Germany (Zunino 1972). Five years later, Kabakov (1977) maintained this synonymisation and added the area of the occurrence of *O. koshantschikoffi* to the distributional range of *O. gibbosus*. Nearly 30 years later, Kabakov (2006) gave this taxon the rank of subspecies—*O. gibbosus koshantschikoffi*—and indicated several morphological characters for distinguishing it; Kabakov (2006) once again demarcated the distributional range of *O. gibbosus koshantschikoffi* while pointing out that it occurs only in the south-eastern part of the species’ range. On the other hand, according to Löbl et al. (2006), Shokhin et al. (2014), and Ziani and Bezděk (2016), this taxon is a separate species, as *Euonthophagus koshantschikoffi*, but these authors gave no comments that would justify the restoration of a specific status for this taxon.

In considering the general distribution of these taxa and the differences in their morphology (Table 2), we support the position of Löbl et al. (2006), Shokhin et al. (2014), and Ziani and Bezděk (2016) that *E. koshantschikoffi* is a valid species and recognize as such. We emphasize, however, that the systematic position of these two taxa requires further research, including study of specimens from their contact zone, preferably using molecular methods.

**Table 2.** Differences in the external morphology between *Euonthophagus gibbosus* and *E. koshantschikoffi*.

**Character**	***Euonthophagus gibbosus* (Fig. 6A–C)**	***Euonthophagus koshantschikoffi* (Fig. 6D–G)**
Head in males	frontal suture arcuately widening towards lateral edges (frontal view), located near clypeus edge (Fig. 6B, C)	frontal suture not widening towards lateral edges (frontal view), located clearly further from clypeus edge, about half of length of head (Fig. 6F, G)
Pronotum	surface matte, rarely with barely noticeable gloss (Fig. 6A)	surface clearly shiny (Fig. 6D, E)
Elytra	surface matte, black, very rarely brown or reddish-brown (in the studied material all of the individuals were uniformly black) (Fig. 6A)	surface slightly shiny, black or reddish-brown (in the studied material approx. 75% of the specimens had a light-coloured elytra) (Fig. 6D, E)
Material examined	64 exx.; Georgia, Armenia, Turkey, Greece, Albania and Spain	55 exx.; Tajikistan (collected in six different localities)

***Euonthophagus sulcicollis* (Reitter, 1892)**

Fig. 4G

Karatag, 30 Jun. 2014, 1058 m a.s.l., [MW] – 2 exx.;

W of Kulob, 3 Jul. 2014, 526 m a.s.l., in dung, [WS] – 8 exx., [MW] – 3 exx.;

Dohanaklik, 16 Jul. 2014, 862 m a.s.l., in dung, [AT] – 2 exx.;

Novabad, 16 Jul. 2014, 580 m a.s.l., [WS] – 1 ex., [AT] – 2 exx.

**Remarks.** The species is widely distributed in Central Asian countries, in Iran and Afghanistan (Kabakov 2006). It was recorded from Kazakhstan, Uzbekistan, Kyrgyzstan, Turkmenistan and Pakistan by Ziani and Bezděk (2016). In Tajikistan, it was noted *inter alia* from Argankun, Vakhshkiy Khrebet, Beshkentskaya Valley, Chiluchor Chashma, Darvaz (Medvedev and Lopatin 1961). This is a rather common species that mainly occurs on loess and sandy soils and it reaches the altitude of 3000 m a.s.l. (Kabakov 2006).

We found 17 individuals in cow dung in rather warm sites in mountain pastures and semi-desert habitats.

**Onthophagus (Altonthophagus) sibiricus Harold, 1877**

Fig. 4H

Takob, 28 Jun. 2014, 2300 m a.s.l., [WS] – 1 ex.

**Onthophagus (Exonthophagus) haroldi Ballion, 1871**

Fig. 4I

Shurroabad, 4 Jul. 2014, 2152 m a.s.l., in dung, [WS] – 1 ex.

**Remarks.***Onthophagus haroldi* is mainly distributed in Central Asia and Kazakhstan, in northeastern Iran, northern Afghanistan, and in the Xinjiang Autonomous Region of China (Kabakov 2006). In Tajikistan, Medvedev and Lopatin (1961) recorded it from the vicinity of Dushanbe, Vakhshkiy Khrebet, Beshkentskaya Valley, and Chiluchor Chashma.

We found a single individual in cow dung on a south-facing grassy slope.

**Figure 6.**
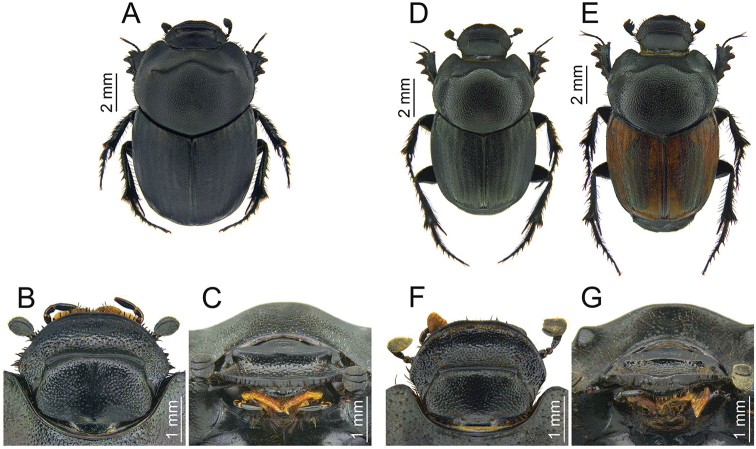
Key characters in two sibling species **A–C***Euonthophagus gibbosus*: **A** habitus **B** head, dorsal view **C** head, frontal view **D–G***Euonthophagus koshantschikoffi*: **D, E** habitus **F** head, dorsal view **G** head, frontal view.

**Onthophagus (Onthophagus) taurus (Schreber, 1759)**

Fig. 7A

Takob, 9 Jul. 2014, 1850 m a.s.l., in dung, [MW] – 6 exx.;

Arykboshi, 2 Jul. 2014, [MW] – 9 exx.;

Karatag, 30 Jun. 2014, 1058 m a.s.l., in dung, [WS] – 3 exx., [MW] – 39 exx., [AT] – 5exx., 17 Jul. 2014, in dung, [MW] – 14 exx., [AT] – 41exx.;

W of Kulob, 3 Jul. 2014, 526 m a.s.l., in dung, [WS] – 1 ex., [MW] – 4 exx., [AT] – 2exx.;

Kangurt, 5 Jul. 2014, 1066 m a.s.l., in dung, [WS] – 2 exx., [MW] – 4 exx.;

Komsomolabad, 11 Jul. 2014, 1160 m a.s.l., in dung, [WS] – 5 exx., [MW] – 6 exx.;

Tojikobod, 13 Jul. 2014, 2223 m a.s.l., [AT] – 5 exx.;

Gharm, 14 Jul. 2014, in dung, [WS] – 3 exx.;

Dohanaklik, 16 Jul. 2014, 862 m a.s.l., in dung, [WS] – 7 exx., [AT] – 4exx.;

Novabad, 17 Jul. 2014, 580 m a.s.l., desert, semi-desert, in cow dung, [MW] – 10 exx., [AT] – 28 exx.

**Figure 7.**
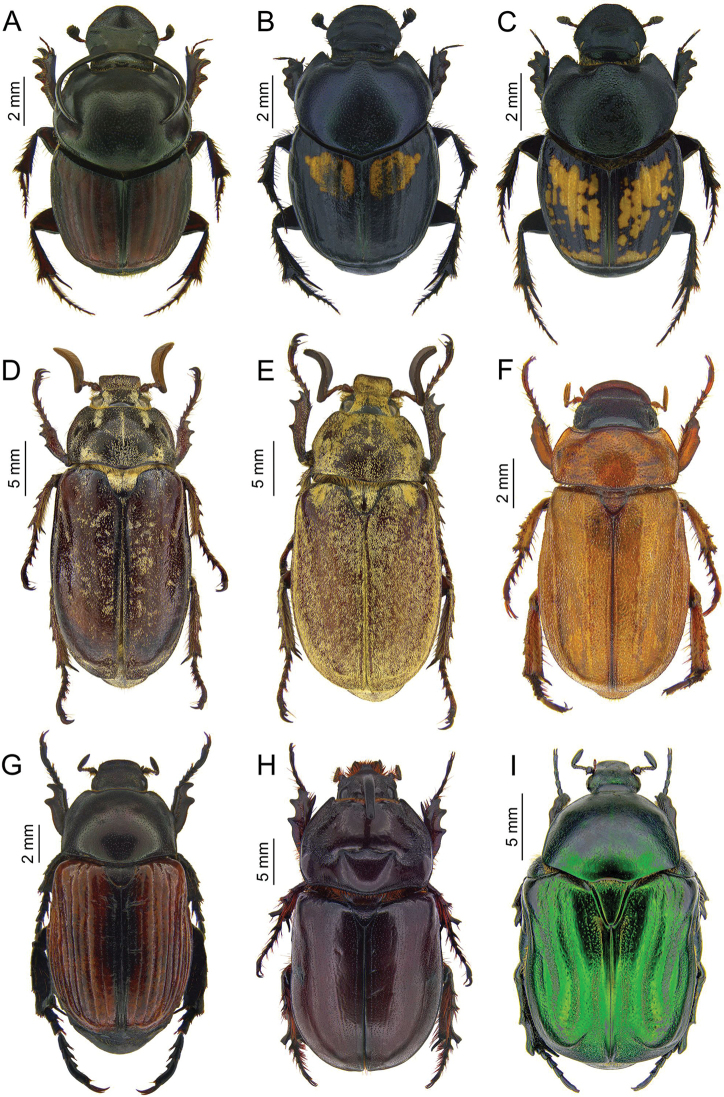
Photos of Scarabaeidae specimens collected during the expedition to Tajikistan in 2014 **A***Onthophagus taurus*, male **B***Onthophagus basipustulatus*, female **C***Onthophagus pygargus*, female **D***Polyphylla tridentata***E***Polyphylla adspersa***F***Adoretus nigrifrons***G***Cyriopertha glabra***H***Oryctes nasicornis turcestanicus***I***Protaetia bogdanoffi*.

**Onthophagus (Palaeonthophagus) afghanus Petrovitz, 1961**

Takob, 9 Jul. 2014, 1850 m a.s.l., in dung, [MW] – 1 ex.;

Karatag, 30 Jun. 2014, 1058 m a.s.l., in dung, [MW] – 2 exx.

**Onthophagus (Palaeonthophagus) basipustulatus Heyden, 1889**

Fig. 7B

Takob, 9 Jul. 2014, 1850 m a.s.l., in dung, [MW] – 1 ex.;

Karatag, 1058 m a.s.l., 30 Jun. 2014, in dung, [MW] – 2 exx.;

Novabad, 17 Jul. 2014, 580 m a.s.l., [MW] – 1 ex.

**Remarks.** This species occurs in the southernmost parts of Kazakhstan, Kyrgyzstan, and mountainous regions of Uzbekistan and Tajikistan; it has also been reported from Afghanistan by Kabakov (2006). Although it has been recorded from Syria, Turkey, Armenia, and Azerbaijan (Ziani and Bezděk 2016), these data most probably relate to another species, *O. formaneki* Reitter, 1897 (Kabakov 2006).

We found four individuals of *O. basipustulatus* in cow dung.

**Onthophagus (Palaeonthophagus) leucostigma (Steven, 1806)**

Sarichashma, 25 Jun. 2014, 921 m a.s.l., in dung, [WS] – 3 exx.

**Onthophagus (Palaeonthophagus) pygargus Motschulsky, 1845**

Fig. 7C

Romit, 26 Jun. 2014, 1250 m a.s.l., [WS] – 1 ex.;

Takob, 9 Jul. 2014, 1850 m a.s.l., in cow dung, [WS] – 5 exx., [MW] – 6 exx., [AT] – 6 exx.;

Arykboshi, 2 Jul. 2014, [MW] – 3 exx.;

Karatag, 30 Jun. 2014, 1058 m a.s.l., in cow dung, [MW] – 6 exx., 17 Jul. 2014, in dung, [MW] – 5 exx.;

W of Kulob, 3 Jul. 2014, 526 m a.s.l., in sheep dung, [MW] – 28 exx.;

Shurroabad, 4 Jul. 2014, 2152 m a.s.l., in horse dung, [WS] – 30 exx., [AT] – 1 ex.;

Novabad, 17 Jul. 2014, 580 m a.s.l., [MW] – 5 exx.

**Remarks.** The species is widespread in Central Asia, from Köpet Dag mountain range in Turkmenistan to the Russian–Chinese border. The northern boundary of its distributional range is limited by the foothills of Tian Shan. It also occurs in Armenia, Iran, Afghanistan, and China (Xinjiang) (Kabakov 2006). In Tajikistan, *O. pygargus* is very common and was recorded from numerous localities including Zeravshanskiy Khrebet, near Marguzor Lake, and Gissarskiy Khrebet, Khozretisho (Medvedev and Lopatin 1961).

We found this species to be one of the most frequently occurring dung beetles in our field survey. We collected it in large numbers in many localities, both in mountain and other pastures near highly urbanized areas. It was found in cow, horse, and sheep dung.

***Scarabaeus carinatus* (Gebler, 1841)**

Ganchi, 25 Jun. 2014, 766 m a.s.l., [WS] – 1 ex.;

Kangurt, 5 Jul. 2014, 1066 m a.s.l., in dung, [MW] – 2 exx., [AT] – 1 ex.

**Remarks.***Scarabaeus carinatus* inhabits mountains of Central Asia and Afghanistan; it is known from Turkmenistan, Uzbekistan, Tajikistan, and Kyrgyzstan. This species has also been recorded from southern and western Kazakhstan (Kabakov 2006) and Iran (Král and Bezděk 2016). It prefers mountain valleys to 3200 m a.s.l. (Kabakov 2006).

We found four individuals in cow dung in mountain pastures.

#### Subfamily Melolonthinae Leach, 1819

**Polyphylla (Mesopolyphylla) tridentata (Reitter, 1890)**

Fig. 7D

Karatag, 30 Jun. 2014, 1058 m a.s.l., at light, [WS] – 1 ex., 17 Jul. 2014, 1058 m a.s.l., at light, [WS] – 2 exx., [LK] – 1 ex.

**Remarks.** The species has been reported from southeastern Uzbekistan, northwestern Tajikistan, and western Kyrgyzstan (Medvedev 1951). In Tajikistan, it is known from the Nauskiy region, Gissarskiy Khrebet, from the vicinity of Lake Iskanderkul, and in the village Obigarm (Medvedev and Lopatin 1961).

In the valley of a mountain river in the vicinity of Karatag, four males were attracted to a UV lamp.

**Polyphylla (Xerasiobia) adspersa (Motschulsky, 1854)**

Fig. 7E

Jilikul, 11 Jul. 2014, 332 m a.s.l., at light, [LK] – 1 ex.;

Arykboshi, 28 Jun. 2014, 906 m a.s.l., at light, [WS] – 2 exx., [LK] – 2 exx., [MW] – 1ex.;

Shahrinav, 1 Jul. 2014, 868 m a.s.l., at light, [WS] – 3 exx., [AT] – 1 ex.;

W of Kulob, 3 Jul. 2014, 537 m a.s.l., at light, [WS] – 1 ex.;

Chavrok, N of Kangurt, 5 Jul. 2014, 1217 m a.s.l., at light, [WS] – 1 ex., [MW] – 1 ex.;

Komsomolabad, 12 Jul. 2014, 1160 m a.s.l., [LK] – 1 ex.

***Amphimallon solstitiale solstitiale* (Linnaeus, 1758)**

Takob, 8 Jul. 2014, 1850 m a.s.l., [WS] – 5 exx., [MW] – 3 exx.;

Shurroabad, 4 Jul. 2014, 2152 m a.s.l., [WS] – 1 ex.;

***Panotrogus myschenkovi* (Ballion, 1871)**

Kangurt, 5 Jul. 2014, 1066 m a.s.l., at light, [AT] – 1 ex.;

Chavrok, N of Kangurt, 5 Jul. 2014, 1217 m a.s.l., at light, [WS] – 2 exx.;

Tojikobod, 13 Jul. 2014, 2223 m a.s.l., [LK] – 1 ex., [MW] – 1ex.

**Maladera (Amaladera) euphorbiae (Burmeister, 1855)**

Jilikul, 24 Jun. 2014, 332 m a.s.l., at light, [AT] – 1 ex.;

Arykboshi, 28 Jun. 2014, 906 m a.s.l., at light, [AT] – 1 ex.;

Karatag, 30 Jun. 2014, 1058 m a.s.l., at light, [LK] – 1 ex.;

Shahrinav, 1 Jul. 2014, 868 m a.s.l., at light [WS] – 1 ex., [AT] – 1 ex. [MW] – 1 ex.

#### Subfamily Rutelinae Macleay, 1819

**Adoretus (Adoretus) nigrifrons (Steven, 1809)**

Fig. 7F

Jilikul, 24 Jun. 2014, 332 m a.s.l., at light, [WS] – 4 exx., [LK] – 2 exx., [AT] – 4 exx., [MW] – 3 exx.;

Arykboshi, 28 Jun. 2014, 906 m a.s.l., at light, [LK] – 3 exx., [AT] – 1ex.;

Karatag, 30 Jun. 2014, 1058 m a.s.l., at light, [LK] – 1 ex., [MW] – 4 exx.;

Shahrinav, 1 Jul. 2014, 868 m a.s.l., at light [WS] – 3 exx.;

W of Kulob, 3 Jul. 2014, 537 m a.s.l., at light, [WS] – 3 exx., [AT] – 1 ex., [MW] – 1 ex.;

Kangurt, 5 Jul. 2014, 1066 m a.s.l., [MW] – 1 exx.;

Chavrok, N of Kangurt, 5 Jul. 2014, 1217 m a.s.l., at light, [WS] – 1 ex., [LK] – 3 exx.;

Gharm, 14 Jul. 2014, meadow, [WS] – 1 ex.

***Anomala oxiana* Semenov, 1891**

Karatag, 17 Jul. 2014, 1058 m a.s.l., at light, [AT] – 1 ex.

**Cyriopertha (Cyriopertha) glabra (Gebler, 1841)**

Fig. 7G

W of Kulob, 3 Jul. 2014, 526 m a.s.l., [MW] – 2 exx.;

Tojikobod, 13 Jul. 2014, 2223 m a.s.l., [WS] – 13 exx., [LK] – 1 ex., [MW] – 3 exx. [AT] – 4 exx.

**Remarks.** This species is distributed in Uzbekistan, northern Tajikistan, southern Kazakhstan, and Kyrgyzstan (Medvedev 1949). It has also been recorded from Eastern Siberia and China (Xinjiang) (Zorn and Bezděk 2016). In Tajikistan, Medvedev and Lopatin (1961) reported it from a number of localities, including Dushanbe, Gissarskiy Khrebet, Fayzabad, Muskinabad, Khozretisho, and Khujand, among others. This species inhabits dry steppes, and it is also often found in farmlands (Medvedev 1949).

We caught this species in mountain meadows, and it was observed in large numbers. Adults were observed flying over the grass and became more active in the evening. It is worth noting that two color forms, the typical brownish form (Fig. 7G) and a melanistic form, were observed sympatrically.

#### Subfamily Dynastinae Macleay, 1819

***Pentodon bidens bidens* (Pallas, 1771)**

Jilikul, 24 Jun. 2014, 332 m a.s.l., at light, [AT] – 1 ex.;

Romit, 26 Jun. 2014, 1283 m a.s.l., at light, [LK] – 1 ex., [MW] – 3 exx.

***Oryctes nasicornis turcestanicus* Minck, 1914**

Fig. 7H

Romit, 26 Jun. 2014, 1283 m a.s.l., at light, [LK] – 2 exx., [MW] – 1 ex.;

Karatag, 30 Jun. 2014, 1058 m a.s.l., at light, [LK] – 1 ex.;

Kangurt, 5 Jul. 2014, 1066 m a.s.l., [MW] – 3 exx.

#### Subfamily Cetoniinae Leach, 1815

**Protaetia (Netocia) bogdanoffi (Solsky, 1876)**

Fig. 7I

Takob, 8 Jul. 2014, 1850 m a.s.l., [WS] – 1 ex., [AT] – 1 ex.;

Chavrok, N of Kangurt, 5 Jul. 2014, 1217 m a.s.l., at light, [WS] – 1 ex.

**Remarks.***Protaetia bogdanoffi* was reported from Uzbekistan, Tajikistan, Kyrgyzstan, and Afghanistan (Medvedev 1964), as well as from Kazakhstan (Bezděk 2016a). In Tajikistan, it has been recorded in Zaalayskiy Khrebet (Trans-Alay Range), Darvaz, Gissarskiy Khrebet, and Khozretisho (Medvedev and Lopatin 1961). This species occurs in open areas, such as steppe and semi-desert habitats, and in river valleys, as well as in higher mountains up to 2500 m a.s.l.

The individuals caught in a mountain meadow in the environs of Takob were collected on flowers, most likely of *Heracleum* or *Prangos*. Another individual, caught at Chavrok, was attracted to a UV lamp near human settlements, in a typical agricultural landscape.

**Protaetia (Netocia) interruptecostata (Ballion, 1871)**

Karatag, 30 Jun. 2014, 1058 m a.s.l., [MW] – 1 ex.

**Protaetia (Potosia) marginicollis (Ballion, 1871)**

Takob, 8 Jul. 2014, 1875 m a.s.l., herbaceous habitat, [AT] – 1 ex., 10 Jul. 2014, alt. 1880 m, [WS] – 1 ex.

Karatag, 1 Jul. 2014, 1088 m a.s.l., shrubs on the river, [AT] – 2 exx.

***Stalagmosoma albellum* (Pallas, 1781)**

Shurroabad, 4 Jul. 2014, 2152 m a.s.l., mountain meadow, pastures, [AT] – 1 ex.

***Oxythyrea cinctella* (Schaum, 1841)**

Sarichashma, 25 Jun. 2014, 921 m a.s.l., [WS] – 2 exx., [LK] – 4 exx., [AT] – 2 exx.;

Romit, 27 Jun. 2014, 1250 m a.s.l., [WS] – 10 exx., [AT] – 1 ex.;

Takob, 9 Jul. 2014, 1850 m a.s.l., [WS] – 2 exx., [AT] – 2 exx., [MW] – 2 exx.;

Arykboshi, 28 Jun. 2014, 906 m a.s.l., [MW] – 3 exx., [AT] – 1ex.;

Karatag, 30 Jun. 2014, 1058 m a.s.l., [WS] – 2 exx., [AT] – 1ex.;

Kangurt, 5 Jul. 2014, 1066 m a.s.l., [WS] – 3 exx.;

Chavrok, N of Kangurt, 5 Jul. 2014, 1200 m a.s.l., [WS] – 4 exx.;

Nurobod, 11 Jul. 2014, 1215 m a.s.l., [WS] – 2 exx., [AT] – 1ex.;

Tojikobod, 13 Jul. 2014, 2223 m a.s.l., [WS] – 2 exx., [LK] – 1 ex.;

Iskanderkul, 18 Jul. 2014, 2300 m a.s.l., meadow, [WS] – 2 exx., [AT] – 1 ex.

## Discussion

Approximately 17% of the scarabaeoid fauna of Tajikistan was recorded during our 25-day survey, which was conducted from 24 June to 18 July 2014. We found one species, *Rhyssemodes transcaspicus*, that has not been previously recorded from the country. The occurrence of three species, *Cheironitis eumenes*, *Onthophagus silus* and *O. ovatus* (Kabakov 2006), had been omitted in the second edition of the Catalogue of Palaearctic Coleoptera (Löbl and Löbl 2016). Moreover, Nikolajev and Pak (2020) described from Tajikistan a new species of the genus *Trochaloschema* (*T. dubium* Nikolajev & Pak, 2020), and Pak and Gubin (2020) described two new species of the genus *Lethrus* (*L. ahriman* Pak & Gubin, 2020 and *L. asmodeus* Pak & Gubin, 2020). Therefore, with these recent publications and our finding of *R. transcaspicus* in Tajikistan, the number of scarabaeoid species in the country is increased to 281.

The most commonly observed species of pleurostict scarab beetles were *Adoretus nigrifrons* (35 exx.) and *Oxythyrea cinctella* (48 exx.). The laparostict scarabs were dominated by: *Onthophagus taurus* (198 exx.), *O. pygargus* (96 exx.), *Euoniticellus fulvus* (116 exx.), and *Euonthophagus koshantschikoffi* (55 exx.). Nineteen species that are typical for the region of Central Asia were also found: Lethrus (Mesolethrus) sp., *Eulasia analis*, *Glaphyrus turkestanicus*, *Gymnopleurus aciculatus*, *Euonthophagus sulcicollis*, *E. koshantschikoffi*, *Onthophagus sibiricus*, *O. haroldi*, *O. afghanus*, *O. pygargus*, *Scarabaeus carinatus*, *Polyphylla tridentata*, *Panotrogus myschenkovi*, *Maladera euphorbiae*, *Anomala oxiana*, *Cyriopertha glabra*, *Protaetia bogdanoffi*, *P. interruptecostata*, and *P. marginicollis*, as well as one subspecies, *Oryctes nasicornis turcestanicus*. Most of the coprofagous Scarabaeoidea of Tajikistan are species whose imagines appear in the spring or at the turn of spring and summer, so the relatively small number of taxa collected is undoubtedly due to the rather late period of our study. Species whose adults start to occur in the summertime constitute less than a half of the scarabeoid beetles of Tajikistan (Medvedev and Lopatin 1961).

Most of the field research on the scarabaeoid beetles of Tajikistan was conducted in the middle of the 20^th^ century and almost exclusively by Russian entomologists. This was partly caused by the political isolation of the country and partly by the lack of transport and tourist infrastructure. After its separation from the Soviet Union in 1991 and the introduction of tourism facilities, the intensification of research was possible for both scientists and amateur entomologists. This, in turn, undoubtedly contributed to a better understanding of the local entomofauna and in a relatively short time resulted in the description of new species and newly recorded taxa for this country (Novikov 1999; Gusakov 2003, 2007, 2008; Nikolajev 2008; Ivanova 2012; Ivanova and Pak 2012; Akhmetova and Frolov 2014).

The richness of the scarabaeoid fauna of Tajikistan and the geography of the country clearly indicate the possibility of finding further species of this superfamily, in particular in near the borders with neighbouring countries where numerous other taxa have been recorded, for example *Eremazus cribratus* Semenov, 1893 (Bezděk 2016b), *Acrossus rufipes* (Linnaeus, 1758), and *Rhodaphodius foetens* (Fabricius, 1787) (Dellacasa et al. 2016). These beetles are rather common in Kazakhstan, Kyrgyzstan, Turkmenistan, or Uzbekistan, and there are suitable similar habitats available for them in Tajikistan. Considering the numerous isolated and largely inaccessible biotopes, the presence of as many as 80 endemic species of Scarabaeoidea, and a remarkably few representatives of the subfamily Aphodiinae, it is very likely to expect some new taxa for the local fauna and perhaps even some yet undescribed. Already, this was confirmed by the recent discovery of three new species of Scarabaeoidea, *Trochaloschema dubium*, *Lethrus ahriman*, and *L. asmodeus*, as well as by the results of studies of other groups of beetles (Greń et al. 2016; Kadyrov et al. 2016) and aphids (Depa et al. 2017). Therefore, new expeditions to Tajikistan are highly desirable.

## References

[B1] Akhmetova LA Frolov AV (2014) A review of the scarab beetle tribe Aphodiini (Coleoptera, Scarabaeidae) of the fauna of Russia. Entomological Review 94(6): 846–879. 10.1134/S0013873814060074

[B2] Bagaturov MF Nikolajev GV (2015) Overview of distribution of the genus *Lethrus* Scopoli, 1777 (Coleoptera: Geotrupidae). Caucasian Entomological Bulletin 11(2): 303–314. [in Russian] 10.23885/1814-3326-2015-11-2-303-314

[B3] Bezděk A (2016a) Subfamily Cetoniinae Leach, 1815. In: Löbl I Löbl D (Eds) Catalogue of Palaearctic Coleoptera.Scarabaeoidea, Scirtoidea, Dascilloidea, Buprestoidea and Byrrhoidea (Vol. 3). Revised and Updated Edition. Brill, Leiden-Boston, 367–412.

[B4] Bezděk A (2016b) Subfamily Eremazinae Iablokoff-Khnzorian, 1977. In: Löbl I, Löbl D (Eds) Catalogue of Palaearctic Coleoptera. Scarabaeoidea, Scirtoidea, Dascilloidea, Buprestoidea and Byrrhoidea (Vol. 3). Revised and Updated Edition. Brill, Leiden-Boston, 98 pp.

[B5] Bezděk A (2016c) Tribe Onitini Laporte, 1840. In: Löbl I Löbl D (Eds) Catalogue of Palaearctic Coleoptera.Scarabaeoidea, Scirtoidea, Dascilloidea, Buprestoidea and Byrrhoidea (Vol. 3). Revised and Updated Edition. Brill, Leiden-Boston, 177–180.

[B6] Dellacasa M Dellacasa G Král D Bezděk A (2016) Tribe Aphodiini Leach, 1815. In: Löbl I Löbl D (Eds) Catalogue of Palaearctic Coleoptera.Scarabaeoidea, Scirtoidea, Dascilloidea, Buprestoidea and Byrrhoidea (Vol. 3). Revised and Updated Edition. Brill, Leiden-Boston, 98–155.

[B7] Depa Ł Kanturski M Taszakowski A Walczak M Bugaj-Nawrocka A Wieczorek K (2017) Dysaphis (Dysaphis) kadyrovi sp. nov.–a new aphid species (Hemiptera: Aphididae) from Tajikistan. Zootaxa 4286(4): 573–585. 10.11646/zootaxa.4286.4.10

[B8] Greń Cz Przewoźny M Szczepański WT Karpiński L (2016) *Dryops renateae* Greń & Przewoźny sp. n. from Tajikistan (Coleoptera: Dryopidae). Zootaxa 4103(2): 177–179. 10.11646/zootaxa.4103.2.8 27394628

[B9] Gusakov AA (2003) New species of the lamellicorn beetles (Coleoptera: Scarabaeoidea: Lucanidae, Scarabaeidae) from the Palaearctic region. Bulletin of the Moscow Society of Naturalists 108(4): 26–30. [in Russian]

[B10] Gusakov AA (2007) A new species of the genus *Netocia* A. Costa, 1852 (Coleoptera: Scarabaeidae: Cetoniinae) from the alpine zone of Tajikistan. Eversmannia 9: 3–7. [in Russian]

[B11] Gusakov AA (2008) New species of genus *Hemictenius* Reitter, 1897 from Tadjikistan. Eversmannia 15–16: 3–5. [in Russian]

[B12] Ivanova YS (2012) A new species of *Lethrus* Scop., 1777 from Tajikistan. The Kharkov Entomological Society Gazette 20(1): 45–47. [in Russian] 10.23885/1814-3326-2012-8-1-21-23

[B13] Ivanova YS Pak OV (2012) A new species of the genus *Trochaloschema* Reitter, 1896 from the central Tajikistan. Caucasian Entomological Bulletin 8(1): 21–23. [in Russian]

[B14] Kabakov ON (1977) A review of scarabeids of the subgenus Euonthophagus Balth. of the genus *Onthophagus* Latr. (Coleoptera, Scarabaeidae) of the USSR and adjacent countries. Revue d’Entomologie de l’USSR 56(2): 383–394.

[B15] Kabakov ON (2006) The lamellicorn beetle subfamily Scarabaeinae (Insecta: Coleoptera: Scarabaeidae) in the fauna of Russia and adjacent countries. Tovarishchestvo Nauchnykh Izdanii KMK, Moskva, 374 pp. [in Russian]

[B16] Kadyrov AKh Karpiński L Szczepański WT Taszakowski A Walczak M (2016) New data on distribution, biology, and ecology of longhorn beetles from the area of west Tajikistan (Coleoptera, Cerambycidae). ZooKeys 606(2): 41–64. 10.3897/zookeys.606.9190 PMC4978010 27551221

[B17] Král D Bezděk A (2016) Tribe Scarabaeini Latreille, 1802. In: Löbl I Löbl D (Eds) Catalogue of Palaearctic Coleoptera.Scarabaeoidea, Scirtoidea, Dascilloidea, Buprestoidea and Byrrhoidea (Vol. 3). Revised and Updated Edition. Brill, Leiden-Boston, 204–207.

[B18] Löbl I Löbl D (Eds) (2016) Catalogue of Palaearctic Coleoptera. Scarabaeoidea, Scirtoidea, Dascilloidea, Buprestoidea and Byrrhoidea (Vol. 3). Revised and Updated Edition. Brill, Leiden-Boston, 983 pp.

[B19] Löbl I Krell FT Ziani S Král D (2006) Tribe Onthophagini Burmeister, 1846. In: Löbl I Smetana A (Eds) Cataologue of Palaearctic Coleoptera.Scarabaeoidea, Scirtoidea, Dascilloidea, Buprestoidea, Byrrhoidea (Vol. 3). Apollo Books, Stenstrup, 159–176. 10.1163/9789004309142

[B20] Medvedev SI (1949) Scarabaeidae. Subfam. Rutelinae. Coleoptera, Vol. 10(3), Fauna of USSR, New series, N 36, 372 pp. [in Russian]

[B21] Medvedev SI (1951) Scarabaeidae. Subfam. Melolonthinae. Part 1. Coleoptera, Vol. 10(1), Fauna of USSR, New series, N 46, 514 pp. [in Russian]

[B22] Medvedev SI (1952) Scarabaeidae. Subfam. Melonthinae. Part 2. Coleoptera, Vol. 10(2), Fauna of USSR, New series, N 52, 276 pp. [in Russian]

[B23] Medvedev SI (1960) Scarabaeidae. Subfam. Euchirinae, Dynastinae, Glaphyrinae, Trichiinae. Coleoptera, Vol. 10(4), Fauna of USSR, New series, N 74, 398 pp. [in Russian]

[B24] Medvedev SI (1964) Scarabaeidae. Subfam. Cetoniinae, Valginae. Coleoptera, Vol. 10(5), Fauna of USSR, New series, N 90, 375 pp. [in Russian]

[B25] Medvedev SI Lopatin IK (1961) Scarab beetles (Coleoptera: Lamellicornia) fauna of Tajikstan and adjacent areas of Central Asia. Trudy Instituta Zoologii i Parazitologii Akademii Nauk Tadzhikskoy SSR 20: 123–148. [in Russian]

[B26] Nikolajev GV (1987) Lamellicornia beetles of Kazakhstan and Middle Asia (Coleoptera, Scarabaeoidea). Nauka, Alma-Ata, 232 pp. [in Russian]

[B27] Nikolajev GV (2003) Coleoptera, Scarabaeidae, Geotrupinae, Lethrini: biology, taxonomy, distribution, key. Kazak Universiteti, Almaty, 254 pp. [in Russian]

[B28] Nikolajev GV (2008) A new species of the genus *Xanthotrogus* Reitter, 1902 (Coleoptera: Scarabaeoidea) from the east part of area distribution. Tethys Entomological Research 16: 23–26. [in Russian]

[B29] Nikolajev GV (2016) Taxonomic composition of the family Trogidae (Coleoptera: Scarabaeoidea) of the Russian fauna. Caucasian Entomological Bulletin 12(1): 81–91. [in Russian] 10.23885/1814-3326-2016-12-1-81-91

[B30] Nikolajev GV Pak OV (2020) A new species of the genus *Trochaloschema* Reitter, 1896 (Coleoptera: Scarabaeidae: Sericinae) from Tajikistan. Caucasian Entomological Bulletin 16(1): 49–52. [in Russian] 10.23885/181433262020161-4952

[B31] Nikritin LM (1973) A review of dung beetles of the genus *Aphodius* from Middle Asia. Entomologičeskoe Obozrenie 52(3): 610–623. [in Russian]

[B32] Novikov OA (1999) A new species of the genus *Trochaloschema* Reitter from the Karateginskiy Mountain Range of Tajikistan. The Kharkov Entomological Society Gazette 7(1): 30–33. [in Russian]

[B33] Pak OV Gubin AI (2020) Two new species of subgenus Furcilethrus Nikolajev, 1968 of genus *Lethrus* Scopoli, 1777 (Coleoptera: Geotrupidae) from Tajikistan. Humanity Space International Almanac 9(5): 547–562. [in Russian] 10.24412/2226-0773-2020-5-547-562

[B34] Protzenko AI (1968) Scarab beetles (Coleoptera, Scarabaeidae) of Kyrgyzstan. Izdatel’stvo “ILIM”. Frunze, 312 pp. [in Russian]

[B35] Rakovič M Král D (2015) Psammodiini (Coleoptera: Scarabaeidae: Aphodiinae: Psammodiini): Supplementary contributions to the first and second editions of the Catalogue of Palaearctic Coleoptera. Folia Heyrovskyana, Series A 23(2): 112–132.

[B36] Rössner E (2018) The Palaearctic species of the genus *Melinopterus* Mulsant, 1842 (Insecta: Coleoptera: Scarabaeidae: Aphodiinae). Vernate 37: 209–306.

[B37] Rössner E Bellmann A (2015) The identity of *Bodilus nigriventris* (Reitter, 1892) and his kinship relations (Coleoptera: Scarabaeoidea: Aphodiidae). Vernate 34: 253–270.

[B38] Safarov N (2003) National strategy and action plan on conservation and sustainable use of biodiversity. Governmental Working Group of the Republic of Tajikistan, Dushanbe, 199 pp.

[B39] Shokhin IV (2002) Scarab Beetles (Coleoptera: Scarabaeoidea) of the Lower Volga Region. In: Komarov E Kalyuzhnaya N Sokhina E (Eds) Biodiversity of Insects of South-Eastern European Part of Russia.Volgograd, 83–137. [in Russian]

[B40] Shokhin IV (2007) Contribution to the fauna of lamellicorn beetles (Coleoptera, Scarabaeoidea) of southern Russia, with some nomenclatural changes in the family Scarabaeidae. Caucasian Entomological Bulletin 3(2): 105–185. [in Russian] 10.23885/1814-3326-2007-3-2-105-185

[B41] Shokhin IV Abdurakhmanov GM Adilkhanova FG (2014) Lamellicorn beetles (Coleoptera, Scarabaeoidea) of the coasts and islands of the Caspian Sea: a survey of the fauna. Ecology of Animals 4: 40–60. [in Russian] 10.18470/1992-1098-2014-4-60-90

[B42] Ziani S Bezděk A (2016) Tribe Onthophagini Burmeister, 1846. In: Löbl I Löbl D (Eds) Catalogue of Palaearctic Coleoptera.Scarabaeoidea, Scirtoidea, Dascilloidea, Buprestoidea and Byrrhoidea (Vol. 3). Revised and Updated Edition. Brill, Leiden-Boston, 180–204.

[B43] Zorn C Bezděk A (2016) Subfamily Rutelinae Macleay, 1819. In: Löbl I Löbl D (Eds) Catalogue of Palaearctic Coleoptera.Scarabaeoidea, Scirtoidea, Dascilloidea, Buprestoidea and Byrrhoidea (Vol. 3). Revised and Updated Edition. Brill, Leiden-Boston, 317–358.

[B44] Zunino M (1972) Revision of the Palaearctic species of the genus *Onthophagus* Latr. (ColeopteraScarabaeoidea). I. The subgenus Euonthophagus Balth. Bollettino del Museo di Zoologia dell’Università – Torino 1: 1–28.

